# OLDER ADULTS REPORTING FEAR OF FALLING PRESENT WITH REDUCED PHYSICAL FUNCTION

**DOI:** 10.1093/geroni/igae098.2719

**Published:** 2024-12-31

**Authors:** Emerson Sebastiao, Vitor Siqueira, Jemimah Bakare

**Affiliations:** University of Illinois Urbana-Champaign, Urbana, Illinois, United States; University of Illinois Urbana-Champaign, Champaign, Illinois, United States; University of Illinois Urbana-Champaign, Champaign, Illinois, United States

## Abstract

Falls pose a significant risk to older adults (OAs), often leading to fear of falling (FOF). This study investigated the prevalence and the impact of FOF on physical activity (PA), sedentary behavior (SB), and physical function in OAs living in a community-dwelling facility (CDF). Ninety-three OAs were included in the study. FOF was assessed using the following Yes/No question: “Are you afraid of falling”? PA and SB were self-reported, while physical function was assessed using a collection of objective tests. Data were analyzed using descriptive and inferential statistics with significance set at p< 0.05. The prevalence of FOF was found to be 47.3% (36.9%–57.9%), with no significant differences between sex (Female: 48.4% (35.8%–61.3%) vs. Male: 44.8% (26.4%–64.3%); χ2(1, N=93) = 0.104, p=0.747) or falls history (zero falls: 43.9% (30.7%–57.6%) vs. One or more falls: 52.8% (35.5%–69.6%); χ2(1, N=93) = 0.704, p=0.401). OAs reporting FOF presented with significant (p< 0.05) lower performance in all physical function tests compared to those reporting no FOF. However, after controlling for the use of assistive devices (i.e., cane), the performance in some tests (i.e., Timed Up and Go, Timed 25-foot Walk; Short Physical Performance Battery subcomponent gait) lost significance (p>0.05). No significant differences were observed for PA and SB between groups with and without FOF (p>0.05). These findings suggest a high prevalence of FOF among the studied sample, worse physical function for OAs reporting FOF, and similar levels of PA and SB between FOF groups of OAs living in a CDF.

